# Plant Copper Amine Oxidases: Key Players in Hormone Signaling Leading to Stress-Induced Phenotypic Plasticity

**DOI:** 10.3390/ijms22105136

**Published:** 2021-05-12

**Authors:** Ilaria Fraudentali, Renato A. Rodrigues-Pousada, Riccardo Angelini, Sandip A. Ghuge, Alessandra Cona

**Affiliations:** 1 Department of Science, University “Roma Tre”, 00146 Rome, Italy; ilaria.fraudentali@uniroma3.it (I.F.); riccardo.angelini@uniroma3.it (R.A.); 2 Department of Life, Health and Environmental Sciences, University of L’Aquila, 67100 L’Aquila, Italy; renatoalberto.rodriguespousada@univaq.it; 3 Interuniversity Consortium National Institute of Biostructures and Biosystems (INBB), 00136 Rome, Italy; 4 The Volcani Center, ARO, Institute of Plant Sciences, Bet Dagan 50250, Israel

**Keywords:** abscisic acid, auxin, copper amine oxidases, hormones, hydrogen peroxide, jasmonic acid, polyamines, root plasticity, wounding

## Abstract

Polyamines are ubiquitous, low-molecular-weight aliphatic compounds, present in living organisms and essential for cell growth and differentiation. Copper amine oxidases (CuAOs) oxidize polyamines to aminoaldehydes releasing ammonium and hydrogen peroxide, which participates in the complex network of reactive oxygen species acting as signaling molecules involved in responses to biotic and abiotic stresses. CuAOs have been identified and characterized in different plant species, but the most extensive study on a *CuAO* gene family has been carried out in *Arabidopsis thaliana*. Growing attention has been devoted in the last years to the investigation of the CuAO expression pattern during development and in response to an array of stress and stress-related hormones, events in which recent studies have highlighted CuAOs to play a key role by modulation of a multilevel phenotypic plasticity expression. In this review, the attention will be focused on the involvement of different AtCuAOs in the IAA/JA/ABA signal transduction pathways which mediate stress-induced phenotypic plasticity events.

## 1. Introduction: The Plant Amine Oxidase Network

Biogenic amines are low molecular weight organic nitrogen compounds, which are present in living cells and include compounds with different structures ranging from aliphatic and aromatic monoamines to diamines and higher polyamines. The diamines putrescine (Put) and cadaverine (Cad), and the polyamines spermidine (Spd) and spermine (Spm) (all together referred as PAs), are aliphatic polycations characterized by the presence of two or more amine functional groups, involved in a lot of physiological events and defense responses in plants [1,2,3,4,5,6,7]. Amine oxidases (AOs) belong to a heterogeneous class of enzymes including copper amine oxidases (CuAOs) and flavin-containing polyamine oxidases (PAOs) and are part of the complex enzyme network catalyzing the oxidative de-amination of PAs to aminoaldehydes. An amine moiety, ammonium and the biologically active hydrogen peroxide (H_2_O_2_), are co-products in the AO oxidation reactions. Plant PAOs oxidize PAs at the secondary amino groups by either a terminal catabolism or interconversion pathway with reaction products depending on both catalytic mechanism and specific substrates. On the other hand, CuAOs are exclusively involved in the terminal catabolism of PAs, oxidizing PAs and monoamines at the terminal amine group with the production of ammonium, the substrate-corresponding aminoaldehyde and H_2_O_2_ [8,9,10,11,12,13,14,15].

## 2. The Amine Oxidase *CuAO* Gene Family: Subcellular Localization and Biochemical Properties of CuAOs from Different Plant Species

CuAOs are homodimeric enzymes consisting of a 70–90 kD subunits, each containing a copper ion and a 2,4,5-trihydroxyphenylalanine quinone (TPQ) cofactor generated by a post-translational autocatalytic modification of an active site tyrosine residue [10,13].

CuAOs have been identified and characterized in different plant species [8,9,10,11,12,13]. The most extensive study on a *CuAO* gene family has been carried out in *Arabidopsis thaliana* (Arabidopsis), in which ten different genes encoding CuAOs have been identified, although only eight of them encode for putative functional CuAOs: *AtCuAOα1* (At1g31670); *AtCuAOα2* (At1g31690); *AtCuAOα3* (At1g31710); *AtCuAOβ* (At4g14940); *AtCuAOγ1* (At1g62810); *AtCuAOγ2* (At3g43670); *AtCuAOδ* (At4g12290); *AtCuAOζ* (At2g42490). The remaining genes, *AtCuAOε1* (At4g12270) and *AtCuAOε2* (At4g12280) are located upstream of *AtCuAOδ* on chromosome 4, and are possibly generated by an *AtCuAOδ* duplication event, followed by an insertion of the transposable element At4g12275 [10,16,17,18,19,20,21]. Recently, eight putative *CuAO* genes (*CsCuAO1*–*CsCuAO8*), distributed on three chromosomes, were identified in *Citrus sinensis* Osbeck genome [22] and a full-length *Camellia sinensis CuAO* gene (*CsCuAO*) was isolated and cloned from a drought tolerant tea cultivar [23]. 

Genetic, biochemical and histological approaches highlight that most of CuAOs localize in the apoplast (AtCuAOβ, AtCuAOγ1, MdAO1, PSAO, ELAO, LSAO, CaCuAO, VfCuAO and NtCuAO [13,14,17,18,24,25,26,27,28,29] or peroxisome (AtCuAOα2, AtCuAOα3, AtCuAOζ, MdAO1, NtDAO1 and NtMPO1 [17,19,25,30] with the exception of AtCuAOδ that has been found to localize in the vacuole [31].

Although CuAOs show a broad variety of substrate specificity, some of them also oxidizing monoamines, CuAOs explored to date primarily oxidize the diamines Put and Cad, while Spd and especially Spm oxidation by most of them is less efficient, with the exception of AtCuAOγ1 and AtCuAOα3, that show an affinity for Spd comparable to that for Put [10,17]. In details, the cell-wall resident CuAOs from *Pisum sativum* (PSAO [11,32]) and *Lens culinaris* (LSAO [11,28,33]) showed a Put/Spd/Spm oxidation ratio of respectively 100:35:0.3 [11] and 100:42:20 [11] while the peroxisomal *Malus domestica* CuAO1 (MdAO1) do not oxidize Spd and Spm, showing activity only with the diamines Put, Cad and 1,3-diaminopropane (Dap) [25]. It is noteworthy that the extracellular *Malus domestica* CuAO2 (MdAO2) exclusively oxidize aliphatic and aromatic monoamines, among which 2-phenylethylamine is oxidatively deaminated to phenylacetaldehyde that in fruits may be converted to 2-phenylethanol, a volatile compound that is a major contributor to fruit flavor and flower fragrance [25]. In *Nicotiana tabacum*, the peroxisomal NtCuAO *N*-methylputrescine oxidase 1 (NtMPO1) that is involved in nicotine biosynthesis, preferentially oxidize *N*-methylputrescine [19,34], while Put oxidation is carried out both in the cell wall and in peroxisome by respectively an apoplastic NtCuAO activity [35] and diamine oxidase 1 (NtDAO1) [19]. The aminoaldehyde produced by the CuAO-driven oxidation of Put is 4-aminobutanal [10], which spontaneously cyclizes to *Δ*^1^-pyrroline that is further converted to *γ*-aminobutyric acid (GABA) by a NAD-dependent aminoaldehyde dehydrogenase. GABA in turn, undergo transamination and oxidation to form succinic acid, which is assimilated into the Krebs cycle, ensuring carbon and nitrogen re-assimilation from Put [11,36,37]. The aminoaldehydes produced by the CuAO-driven oxidation of Cad and Spd are 5-aminovaleraldheyde and 3-aminopropyl-4-aminobutyraldehyde, spontaneously cyclizing to *Δ*^1^-piperideine or 1,5-diazabicyclononane, respectively [14]. 

## 3. CuAOs in Development and Hormonal Signaling Pathways: Analysis of Tissues Specific- and Hormone/Stress-Modulated-CuAO Expression Profiles

**Figure 1:**
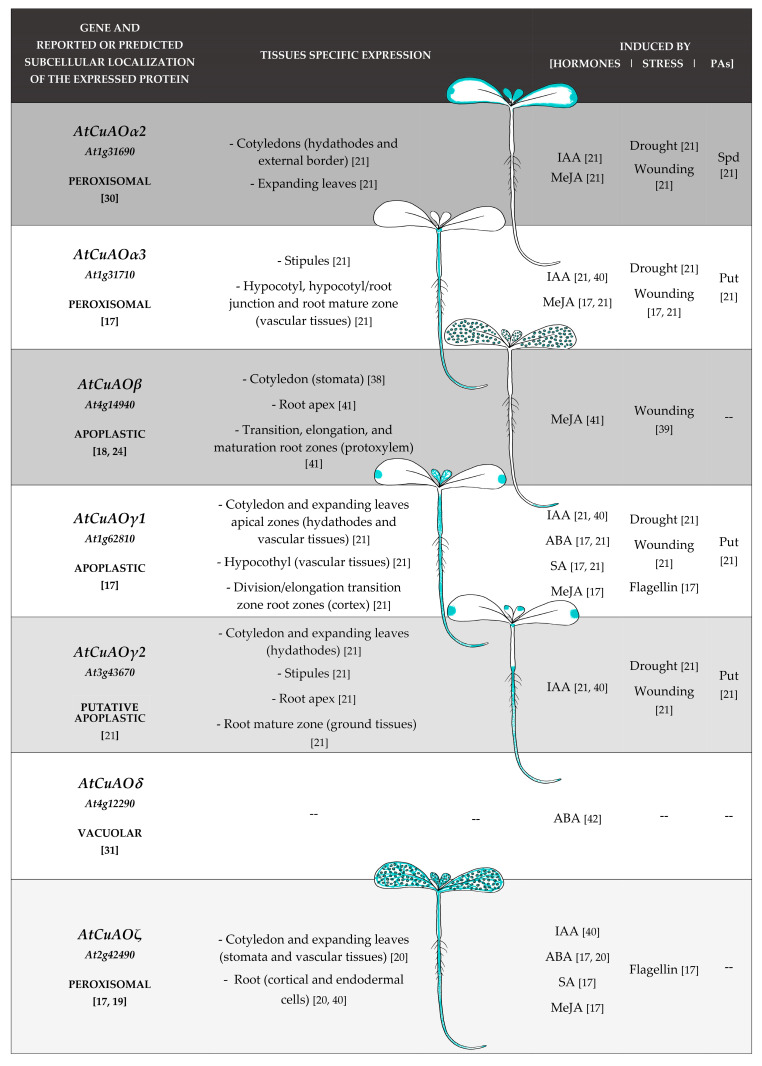
Schematic representation of transcriptional activity of *CuAO* promoters in organs/tissues of Arabidopsis plantlets and induction by stress, stress-related hormones and PAs.

Growing attention has been devoted in the last years to the investigation of the CuAO expression pattern during development and in response to an array of stress and stress-related hormones. To this purpose, tissue-specific expression analysis and quantitative expression studies have been performed exploiting *AtCuAOs promoter::green fluorescent protein-β-glucuronidase fusion* (GFP-GUS) transformed Arabidopsis plants and RT-qPCR approaches. In Arabidopsis seedlings at early developmental stage, *AtCuAO* promoter activity was revealed in tissues involved in water supply and/or water loss such as hydathodes, stomata and vascular tissues as well as in growing tissues responsible for organ development and growth rate modulation. Expression has been found in hydathodes of expanding leaves (*AtCuAOγ1* and *AtCuAOγ2* [21]) and/or cotyledons (*AtCuAOα2*, *AtCuAOγ1* and *AtCuAOγ2* [21]); in stomata of cotyledons (*AtCuAOβ* [38]; *AtCuAOζ* [20]) and flowers (*AtCuAOβ* [38]) as well as in vascular tissues of (*i*) cotyledons, expanding leaves and hypocotyl (*AtCuAOγ1* [21]); (*ii*) hypocotyl, hypocotyl/root junction and root mature zone (*AtCuAOα3* [21]); (*iii*) root transition, elongation, and maturation zones (*AtCuAOβ* [39]); (*iv*) cotyledons and expanding leaves (*AtCuAOζ* [40]). Moreover, *AtCuAO* expression has been found in cotyledon external border and in expanding leaves (*AtCuAOα2* [21]); in stipules (*AtCuAOα3* and *AtCuAOγ2* [21]); in the cortex of the division/elongation transition root zone (*AtCuAOγ1* [21]); root apex (*AtCuAOβ* and *AtCuAOγ2* [21]); ground tissues of root mature zone (*AtCuAOγ2* [21]); root cortical end endodermal cells (*AtCuAOζ* [40]) (Figure 1).

Analysis of PA- modulated expression profiles of *AtCuAOs* reveals that most of the characterized *AtCuAOs* are induced by Put (*AtCuAO α2*/*α3/γ1*/*γ2*) [21] (Figure 1). On the contrary, expression of *AtCuAOs* may be either induced (*AtCuAOα2*/*α3*) or inhibited (*AtCuAOα3*/*γ1*) by Spd, depending on treatment duration for *AtCuAOα2* [21].

Analysis of hormone- and stress-modulated expression profiles of *AtCuAOs* reveals that most of the characterized *AtCuAOs* are modulated by stress and/or by stress-related hormones. As shown in Figure 1, *AtCuAOα2*/*α3*/*γ1*/*γ2*/*β* are all induced by wounding and methyl-jasmonate (MeJA) [17,21,38,39] with the exception of *AtCuAOγ2* that is inhibited by MeJA treatment [21], four of them by drought and indole-3-acetic acid (IAA) (*AtCuAOα2*/*α3*/*γ1*/*γ2* [21,40]), while *AtCuAOβ* is not responsive to IAA [41]. Interestingly, after both treatments a tardive repression occurs after the early reported gene induction for *AtCuAOα2*/*α3*/*γ2* (IAA treatment) and *AtCuAOγ2* (drought) [21]. *AtCuAOζ* expression is also induced by IAA and MeJA [17,40], while it is repressed by wounding [17]. Moreover, abscisic acid (ABA), flagellin and salicylic acid (SA) are all active in inducing both *AtCuAOγ1* and *AtCuAOζ* expression [17,20], while ABA was reported to induce *AtCuAOδ* [42]. However, *AtCuAOα2*/*α3*/*γ2* are repressed by both ABA and SA, while *AtCuAOγ1* shows an early inhibition of gene expression preceding the SA reported induction [21]. Data about wounding and MeJA effect on AtCuAO expression, are in line with previous evidences reporting the induction of CuAO expression in *Hordeum vulgare* [43] and *Nicotiana tabacum* [44] upon MeJA treatment as well as in *Cicer arietinum* upon wounding and jasmonic acid (JA) treatment [45].

## 4. CuAOs as PA Level Modulators and Sources of Reactive Compounds in the Cytoplasm and Cell Wall

Many studies in different plant species highlight the contribution of AOs to plant development and defence responses by a dual role, as PA level modulators and bioactive compound sources, that is H_2_O_2_ and aminoaldehydes. Considering that both PAs and H_2_O_2_ may trigger signal transduction pathways leading to phenotypic plasticity or defense responses during developmentally controlled or stress-induced events, AO may represent a checkpoint in the balance of the PAs/H_2_O_2_ ratio, supposed to be responsible for important physiological events such as cell fate determination in developmental, or hypersensitive (HR), cell death [46].

PA-derived aminoaldehydes represent important metabolites involved in carbon and nitrogen recycling and/or redirecting to different metabolic pathways, as for instance Put-/methylPut-derived aminoaldehydes, respectively precursors of GABA and alkaloids, which have been shown to rapidly accumulate in stressed plants. In this regard, CuAO activity has been suggested to contribute via PA-derived aminoaldehyde (*i*) in salt stress-induced GABA accumulation in *Glycine max* [47], (*ii*) in wound-induced GABA accumulation in *Pisum sativum* [37], (*iii*) in MeJA-induced nicotine biosynthesis in *Nicotiana tabacum* [19] and (*iv*) to the production of 2-phenylethanol, responsible for fruit flavour and flower fragrance in *Malus domestica* [25]. 

CuAOs also participate in signalling processes via regulation of nitric oxide (NO) production. In this regard, noteworthy is the involvement of apoplastic AtCuAOγ1 in ABA-mediated stress responses via ABA-induced NO production [48], CuAO activity-derived H_2_O_2_ possibly acting as an upstream trigger for NO biosynthesis [48]. Moreover, the peroxisomal AtCuAOα2 has been proposed to have a role in arginine-dependent NO synthesis by altering arginine availability through modulation of arginase activity [16,49]. 

PA homeostasis may assume a key role in rapidly proliferating and fast growing tissues involved in organ formation, in which regulation of PA cellular content is implicated in cell cycle regulation. CuAOs play a role at the early cell division phase of tuber growth in *Helianthus tuberosus* [50], in cell growth cycle at the onset of cell division [51] and in cell cycle-endocycle progression in vascular tissues in *Nicotiana tabacum* [52]. The occurrence of AtCuAOs in meristematic zones, where an auxin maximum is positioned, has been observed [21]. Recently, PA homeostasis accomplished by AtCuAOδ has been revealed to take part in GA-mediated control of germination, leaf development and flowering time by a reduction in GA biosynthesis. Concerning this, the change in auxin levels may represent a possible causal link between the increase in Put level in *Atcuaoδ* mutants and the reduction in expression of GA biosynthesis genes [53]. Likewise, perturbation of CuAO/PAO-mediated maintenance of PA homeostasis, by unbalancing PA biosynthesis/oxidation rates, may contribute to PA accumulation under osmotic stress in *Vitis vinifera* cv. Chardonnay, during which impaired defense responses against pathogens and reduced ABA biosynthesis have also been observed [54]. This evidence emphasizes the role of PA oxidation, via either PA homeostasis or H_2_O_2_ delivering, in osmotic stress signaling and defense responses upon biotic stress and suggests a link between PA oxidation and ABA biosynthesis under osmotic stress [54].

In this regard, a central role has been ascribed to H_2_O_2_ derived from PA oxidation, especially regarding the cell wall-resident AOs whose activity is restricted by the limited amounts of extracellular PAs and depends on developmentally regulated or stress-induced PA secretion [35,46,55,56]. The absence/low amount of PAs in the apoplast, along with the occurrence of high AO levels in the apoplast also in non-stressing environments [56], suggest a role of wall-secreted PAs mainly as H_2_O_2_ sources by their AO-driven oxidation rather than signalling compounds themselves. In the cell wall, H_2_O_2_-derived from CuAO-mediated PA oxidation may represent a significant part of the ROS apoplastic network and behaves as a multifunctional compound: (*i*) triggering intracellular signal transduction pathways leading to increased expression of defence genes and hypersensitive (HR)-cell death in infected area; (*ii*) playing a role in defense against plant pathogens by a direct anti-microbial activity and (*iii*) participating as co-substrate in peroxidase-mediated lignin biosynthesis and cross-linking of cell wall polysaccharides and proteins in the modulation of cell wall expansion and its maturation after cessation of growth [11,12,13]. Accordingly, CuAO-driven H_2_O_2_ production in the apoplast has been implicated in cell wall stiffening events responsible for developmental cell wall maturation in *Pisum* sativum [26], *Lens culinaris* [26], *Cicer arietinum* [26,45] and *Nicotiana tabacum* [52], lignin deposition in differentiating protoxylem elements in Arabidopsis [18,41] as well as during defence responses to pathogen attack and wound healing in *Cicer arietinum* [45]. The apoplastic NtCuAO-driven H_2_O_2_ production has been involved in early root xylem differentiation in *Nicotiana tabacum* plants over-expressing a fungal endopolygalacturonase, which are characterized by constitutively activated defense responses [57]. Similarly, the apoplastic AtCuAOβ-driven H_2_O_2_ production has been shown to be a key factor in leaf wounding-induced JA-mediated early root protoxylem differentiation in Arabidopsis [39,41]. Noteworthy, H_2_O_2_ derived from the apoplastic VfCuAO-mediated oxidation of Put has been reported to be involved in ABA-induced and darkness-induced stomatal closure in *Vicia faba* through a mechanism involving Ca^2+^ as a second messenger [20,29,58]. Furthermore, apoplastic AtCuAOγ1-driven Put oxidation elicits ROS-dependent SA-signaled pathways leading to activation of plant defenses in Arabidopsis [59].

Concerning intracellular CuAO-derived H_2_O_2_, the peroxisomal AtCuAOζ [20] and the vacuolar AtCuAOδ [42] have been reported to be involved in ABA-induced stomatal closure through H_2_O_2_ production.

Overall, recent literature highlight CuAOs to play a key role in ontogenetic and environment-regulated developmental events as well as in defence responses by modulation of a multilevel phenotypic plasticity expression, ranging from fast stomatal closure or metabolic plasticity to produce stress-related compounds, up to long-term phenotypic acclimation including organ growth rate, photoperiod/seasonal-dependent events and morpho-anatomical traits. Hereafter, the attention will be focused on the role of the different AtCuAOs in stress-induced phenotypic plasticity as components of the IAA/JA/ABA signal transduction pathways (Table 1). 

## 5. AOs Play a Role in Xylem Phenotypic Plasticity

It is known that xylem vessels differentiation depends on coordinated events of cell wall lignification and developmental PCD, both requiring H_2_O_2_. In this regard, it has been suggested that the H_2_O_2_ produced by cell-wall localized AOs may play a key role in the maturation of xylem cells by triggering the peroxidase-driven polymerization of monolignols and by signaling developmental PCD in differentiating xylem precursors, depending on developmentally regulated or stress-induced PA secretion into the apoplast [13,41,57,60,61]. In physiological conditions, xylem differentiation [62,63] and root meristem size [64,65] are respectively controlled by the auxin/cytokinin/Thermo-Spm and auxin/cytokinin loops [62,63], which integrate the vascular patterning in the root developmental program, strictly coordinating root length, meristem size and protoxylem element position [66]. However, under stress conditions this correlation may be disrupted, and meristem size and protoxylem position may vary independently of each other [66]. Intriguingly, ROS, especially the O_2_^−^/H_2_O_2_ ratio, can also affect root meristem size and transition from cell proliferation to differentiation independently of the auxin/cytokinin pathway [67,68]. In this regard, the ROS-dependent signaling pathway responsible for meristem size positioning involves UPBEAT transcription factors repressing peroxidase expression in the elongation zone [67]. Likewise, under stress conditions, the dominance of auxin/cytokinin/Thermo-Spm loop in xylem differentiation could be overwhelmed by different signals and xylem differentiation has been suggested to be triggered by stress-induced AO-driven H_2_O_2_ production [39,41,57,60,69]. On the basis of these results, it is tempting to hypothesize that root xylem differentiation in normal conditions is under the auxin/cytokinin/T-Spm loop dominance, as well specific modulation of peroxidases, or other oxidases such as AOs, involved in ROS homeostasis, that regulate the timing of the differentiation events and the correct position of the xylem elements with respect to the apex. However, under stress conditions, such as those simulated by PA treatment [60] and endopolygalacturonase over-expression [57], as well as those mediated by JA [41], an increased stress-induced AO expression and/or PA secretion result in enhanced production of H_2_O_2_ that may become the prevalent signal leading to early protoxylem precursors differentiation. 

## 6. Expression of IAA- and Stress-Inducible AtCuAOs in Free-Auxin Maximum Zones Suggest an Role in Both Developmentally Regulated and Stress-Induced Xylem Differentiation in the Leaf and in the Root

Leaf vascular differentiation occurs under hormonal control at early stages of primordium development. To explain vascular patterning in dicotyledonous leaves, the *leaf venation hypothesis* has been proposed by which an auxin gradient arising from the free-auxin maximum zones induces xylem vessels differentiation, depending on the distance between the site of hormone synthesis and the location of the differentiating vascular tissues [70,71,72]. Fast-growing regions are the major locations of auxin production, especially hydathodes, which develop in the leaf tip and later in the lobes and represent primary sites of auxin synthesis during leaf morphogenesis, and stipules near the shoot apex [72]. During leaf primordium development, free-auxin maximum zones gradually shift basipetally along the margins up to the central leaf region. 

**Figure 2:**
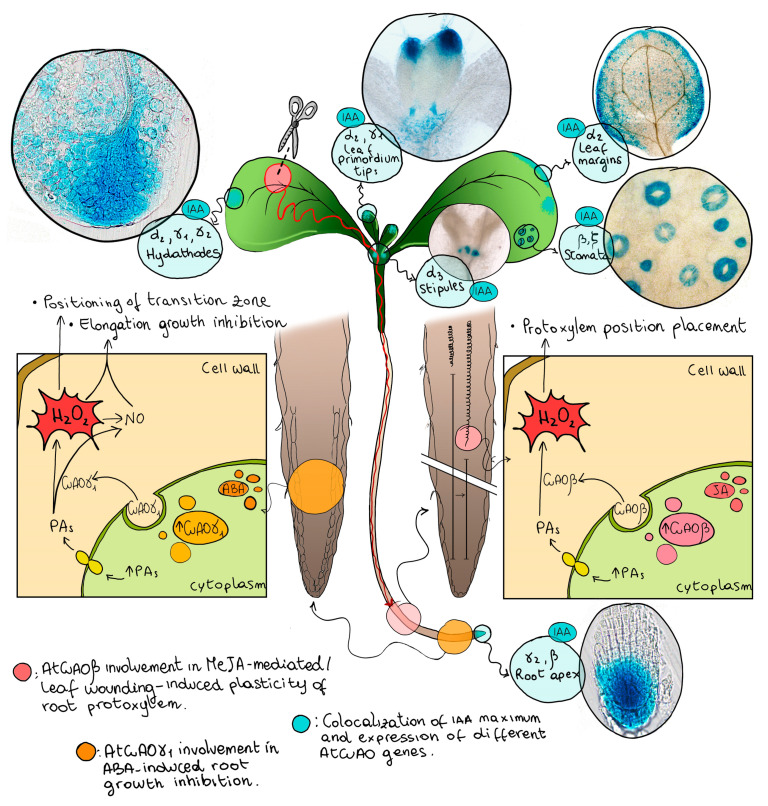
Involvement of AtCuAOs in long distance signaling and stress-induced phenotypic plasticity. Pink circles: schematic representation of AtCuAOβ involvement in MeJA-mediated/leaf wounding-induced plasticity of root protoxylem. Leaf-wounding triggers a long-distance leaf-to-root signaling leading to MeJA-mediated/AtCuAOβ-driven early root protoxylem differentiation, highlighted by changes in root protoxylem position (i.e., the distance from the root apical meristem of the first protoxylem cell with fully developed secondary wall thickenings) [39,41]. Blu circles: co-localization of [IAA] maximum and the expression of *AtCuAO* genes. Images associated to [IAA] maximum show GUS staining microscopy analysis of *AtCuAOα2*/*α3*/*γ1*/*γ2*/*β* promoter::green fluorescent protein-β-glucuronidase fusion (prom-*AtCuAO::GFP-GUS*) transgenic plants (unpublished images; transformed plants and experimental conditions have been described in [38,39]). Representative images for each tissue/organ are shown. Orange circles: A schematic representation of *AtCuAOγ1* involvement in ABA-induced root growth inhibition by elongation growth regulation and transition zone positioning [48].

Noteworthy, it has been reported that expression of some members of the *AtCuAO* family is IAA-inducible (*AtCuAOα2*/*α3/ γ1*/*γ2*/*ζ*) and show a tissue specific expression in zones where a free-auxin maximum has been reported [21,72,73]. Hereafter, the overlap between the expression of members of the *AtCuAO* family and free-auxin maximum zones is summarized (Figure 2). *AtCuAOα3* is expressed in stipules [21]; *α2* and *γ1* in leaf primordium tips [21]; *α2, γ1* and *γ2* in hydathodes [21]; *α2* in leaf margins of expanding leaves or cotyledons [21]; *γ2* in the root apex [21] and *AtCuAOβ* and *AtCuAOζ* in stomata [20,38]. Considering these data, it is reasonable to hypothesize that the peroxisomal AtCuAOα2/α3/ζ and the apoplastic γ1/γ2 contribute to developmentally regulated xylem differentiation in leaf (α2/α3/γ1/γ2/ζ) and root (γ2), by H_2_O_2_ production in the apoplast and in the peroxisome and/or PA homeostasis in the peroxisome. Moreover, the evidence that the expression of IAA-inducible *AtCuAOα2*/*α3*/*γ1*/*γ2*/*ζ* are also inducible by different stress and stress-related hormones (Figure 1 and Figure 2), allows to hypothesize that their expression in areas of free auxin production responsible for xylem differentiation patterning, could be also suitable for xylem adaptive plasticity under stress conditions in the leaf as well in the root, independently from the auxin/cytokinin loop, such as during drought stress to enhance water uptake by improving root hydraulic conductivity.

## 7. The AtCuAOβ-Driven H_2_O_2_ Production Plays a Role in Stress-Induced/MeJA-Signaled Early Protoxylem Differentiation in the Root

A key role has been ascribed to *AtCuAOβ* in root xylem differentiation under both biotic stress, such as nematode infection, and abiotic stress as well as abiotic-simulated stress conditions, such as wounding and MeJA-signaled wound healing. 

It has been reported that the cell wall-localized AtCuAOβ is expressed in correspondence of free-IAA maximum sites, such as root cap cells and protoxylem precursors at early stages of vascular tissue differentiation, and that its expression profile overlaps with lignin biosynthesis [18,41]. In particular, the AtCuAOβ role in extra-cellular cross-linking of structural proteins or lignin precursors has been demonstrated during interactions with nematode parasites, correlating the *AtCuAOβ* profile expression with the re-differentiation process of root vascular tissues which occurs to contrast the effects of mechanical pressure caused by the nematode [74]. Moreover, it has been shown that *AtCuAOβ* expression is strongly induced by the wound-signal MeJA, especially in protoxylem at the transition, elongation and maturation zones [41]. Furthermore, it has been also reported that MeJA-treatment induces an early root protoxylem differentiation along with H_2_O_2_ accumulation in the position where the first cell with fully developed cell wall thickening is detected, while it does not occur in *Atcuaoβ* loss-of-function mutants [41]. The absence of any detectable phenotypes in *Atcuaoβ* mutants and the ineffectiveness of IAA in inducing *AtCuAOβ* expression [41] strongly suggest a peculiar role of this *AtCuAO* in stress-induced protoxylem plasticity in root.

## 8. AtCuAOβ Is Involved in Early Root Protoxylem Differentiation Induced by Leaf Wounding through Systemic Signaling

A complex system for regulating growth and development is found in plants to deal with a plethora of external stimuli and continuous climate changes. Long-distance signaling is an essential leaf-root communication pathway, as it quickly and effectively triggers distal systemic response to environmental changes or mechanical damage perceived locally in specific plant areas [75]. When tissue damage occurs, the wound site become an easy way for pathogen infection and water loss and triggers both local responses, leading to wound healing [57,76,77], and systemic responses, through a complex signaling pathways which spread the damage message from the injured tissues through the whole plant [75]. The sensing of wounding includes the production in the damaged area of early signaling molecules, among which the most important are ROS, glutamate and damage associated molecular patterns (DAMP), such as oligogalacturonides [76]. Glutamate triggers an increase of intracellular Ca^2+^ level through the activation of glutamate receptor-like proteins (GLRs), which are cation-permeable channels. Cytosolic Ca^2+^ increase is propagated as a wave to adjacent tissues leading to the systemic induction of JA biosynthesis at distal sites from the wound [78]. JA signaling in distal sites induces defense responses and the expression of stress-related genes among which *AOs* [45].

In this context, the occurrence of a H_2_O_2_-mediated root phenotypic plasticity triggered through long-distance signaling pathways travelling from the wounded-leaf to the root has been reported. In detail, a specific H_2_O_2_ accumulation in root protoxylem has been detected soon after the injury followed by early root–protoxylem differentiation a few days later, with no observable changes in meristem size and whole root growth [39,69]. The effects of leaf wounding on H_2_O_2_-induced protoxylem differentiation in root along with the hormonal regulation features of *AtCuAOβ* expression, that is induced by MeJA and is un-responsive to IAA treatment, suggest that, under stress conditions caused by distal wounding, an extracellular H_2_O_2_ production may drive early root protoxylem differentiation independently from the auxin/cytokinin/T-Spm loop occurring in normal-growth condition. The involvement of the H_2_O_2_ delivered from AtCuAOβ-mediated PA oxidation in the early root protoxylem differentiation which occurs after leaf wounding [39], provides a link between an abiotic stress and a distal root phenotypic plasticity via systemic signaling (Figure 2).

As a whole, since JA level increases in drought-stressed tissues and dehydration induces both the expression of many wound-related genes and modifications of xylem position, it is possible that leaf wounding activates dehydration signaling in roots leading to systemic event of phenotypic plasticity. In the specific case of the MeJA-mediated leaf wounding-induced early xylem differentiation in Arabidopsis root, the stress-signaling molecule H_2_O_2_ derives from the oxidation of PAs mediated by the apoplastic AtCuAOβ [39,41,61]. In this context, root xylem plasticity could represent a rapid response directed to both increase water supply and remedy water loss consequent to the injury.

## 9. AtCuAOγ1 Is Involved in ABA-Associated Growth Responses

The stress related-hormone ABA is well known to be involved in stomatal closure, maintenance of seed dormancy and rhizogenesis induced by drought. While the biosynthetic pathway of ABA is well known, its signaling mechanisms are complex and involve a large number of factors. A link between AO and nitric oxide (NO) in ABA-mediated stress responses, like the ABA-induced inhibition of germination and root growth, has been shown [48]. Plant growth and development, senescence, flowering, abiotic stress responses including stomatal closure and defense responses to pathogens are some of the various physiological processes in which both AOs and NO are involved, and these overlapping roles led to hypothesizes an interaction in their signaling pathways. In Arabidopsis seedlings, NO biosynthesis is induced by treatment with PAs through a pathway in which AOs are supposed to be directly or indirectly involved [79]. In details, insertional mutants deficient in AtCuAOγ1 (*Atcuaoγ1*) have been shown to be defective in NO production and altered in both root growth and germination potential after treatment with ABA [80]. Consistently with a role of AtCuAOγ1 in ABA-induced NO-mediated root growth inhibition, *AtCuAOγ1* expression is induced by ABA, especially in the root cortex of the root transition/elongation zone [21] (Figure 2). However, CuAOs do not directly produce NO, but may alter NO levels indirectly via H_2_O_2_ derived from CuAO-mediated PA oxidation, that opens some calcium channels, supposed to act upstream of NO biosynthesis [81,82].

## 10. Conclusions

This review updates knowledge on the physiological functions of plant CuAOs focusing on xylem phenotypic plasticity. In the first part of this review, a comprehensive analysis of subcellular localization, biochemical properties and hormone/stress-modulated expression profiles of CuAOs from different plant species is reported, with a special focus on AtCuAO tissue-specific expression. In the second part, the dual role of CuAOs on PA homeostasis and delivering of reactive/signaling compounds is argued. In particular, the main focus of this review concerns the role of CuAOs as component of local or systemic signaling pathways leading to developmentally regulated or stress-induced xylem phenotypic plasticity. 

Auxin is a key regulator of vascular differentiation both in leaves, according to the *leaf venation hypothesis*, and roots, according to auxin/cytokinin/Thermo-Spm loop. The IAA-inducible expression of some members of the *AtCuAO* family in zones where a free-auxin maximum has been reported, leads to hypothesize that this class of enzymes may contribute to developmentally regulated xylem differentiation by H_2_O_2_ production, both in leaves and roots. Furthermore, since *AtCuAO* expression is also inducible by various stresses or stress-related hormones, it is tempting to speculate that their expression in free auxin production areas responsible for the xylem differentiation, could also be involved in events of xylem plasticity required for adaptation under stress conditions.

Indeed, it has been shown that the AtCuAOβ-driven H_2_O_2_ production plays a role in stress-induced/MeJA-signaled early protoxylem differentiation in the root. Furthermore, AtCuAOβ is involved in long-distance leaf-to-root signaling pathways leading to early root protoxylem differentiation upon leaf wounding. Overall, this review highlights the CuAO role in stress-induced phenotypic plasticity, especially early root protoxylem differentiation.

## Figures and Tables

**Table 1:** Subcellular localization and physiological functions of plant CuAOs.

CuAOs/Plant Sources	Subcellular Localization	Reported Physiological Functions
**AtCuAOβ** **Arabidopsis**	Apoplast [18,24]	MeJA-induced vascular development via PA-derived H_2_O_2_ [18] Leaf wounding-induced vascular development via PA-derived H_2_O_2_ [39]
**AtCuAOα2** **Arabidopsis**	Peroxisome [30]	Salt stress/elicitor-induced NO production affecting primary root growth via arginase activity down-regulation [16]
**AtCuAOγ1** **Arabidopsis**	Apoplast [17]	ABA-mediated inhibition of germination and root growth via NO induced by PA-derived H_2_O_2_ [48] SA-signaled pathways leading to activation of plant defenses against pathogens via PA-derived H_2_O_2_ [59]
**AtCuAOδ** **Arabidopsis**	Vacuole [31]	ABA-mediated stomatal closure via PA-derived H_2_O_2_ [42] GA-mediated development (germination, leaf development and flowering time) via PA homeostasis [53]
**AtCuAOζ** **Arabidopsis**	Peroxisome [17,19]	ABA-induced stomatal closure via PA-derived H_2_O_2_ [20] IAA-induced lateral root development via PA-derived H_2_O_2_ [40]
**MdAO1** ***Malus domestica***	Peroxisome [25]	GABA production via PA-derived aminoaldehyde [25]
**MdAO2** ***Malus domestica***	Apoplast [25]	Deamination of 2-phenylethylamine for 2-phenylethanol production (a contributor to fruit flavour and flower fragrance) [25]
**PSAO** ***Pisum sativum***	Apoplast [14,26]	Lignification and/or wall-stiffening events in plant growth and development via PA-derived H_2_O_2_ [26] Wound-induced GABA accumulation via PA-derived aminoaldehyde and H_2_O_2_ –driven lignification [37]
**ELAO** ***Euphorbia characias***	Apoplast [27]	-
**LSAO** ***Lens culinaris***	Apoplast [14,26,28]	Lignification and/or wall-stiffening events in plant growth and development via PA-derived H_2_O_2_ [26]
**CaCuAO** ***Cicer arietinum***	Apoplast [14,26]	Lignification and/or wall-stiffening events in plant growth and development via PA-derived H_2_O_2_ [26] Wound healing and defense responses to pathogen via PA-derived H_2_O_2_ [45]
**GmCuAO** ***Glycine max***	-	Salt stress-induced GABA accumulation via PA-derived aminoaldehyde [47]
**HvCuAO** ***Hordeum vulgare***	-	MeJA-mediated protection against powdery mildew fungus *Blumeria graminis* infection [43]
**HtCuAO** ***Helianthus tuberosus***	-	Role in development at the early cell division phase of tuber growth and during the cell enlargement [50]
**VfCuAO** ***Vicia faba***	Apoplast [29]	ABA-induced stomatal closure via PA-derived H_2_O_2_ [20,29] Dark-induced stomatal closure via PA-derived H_2_O_2_ [58]
**NtCuAO** ***Nicotiana tabacum***	-	Cell growth cycle at the onset of cell division via PA homeostasis [51] Cell cycle-endocycle progression in vascular tissues [52] Defense response to pathogen via apoplastic PA-derived H_2_O_2_ [35] Early root xylem differentiation via apoplastic PA-derived H_2_O_2_ [57]
**NtDAO1** ***Nicotiana tabacum***	Peroxisome [19]	PA (Put) catabolism [19]
**NtMPO1** ***Nicotiana tabacum***	Peroxisome [19]	*N*-methylated Put as substrate in MeJA-induced alkaloid biosynthesis [19,34]
